# RIPK3-Mediated Necroptosis and Apoptosis Contributes to Renal Tubular Cell Progressive Loss and Chronic Kidney Disease Progression in Rats

**DOI:** 10.1371/journal.pone.0156729

**Published:** 2016-06-09

**Authors:** Yongjun Zhu, Hongwang Cui, Yunfeng Xia, Hua Gan

**Affiliations:** 1 Department of Nephrology, the First Affiliated Hospital of Chongqing Medical University, Chongqing, China; 2 Department of Nephrology, the Affiliated Hospital of Hainan Medical College, Haikou, China; 3 Department of Orthopedics, the First Affiliated Hospital of Chongqing Medical University, Chongqing, China; Toho University School of Medicine, JAPAN

## Abstract

Tubulointerstitial fibrosis (TIF) is caused by the progressive loss of renal tubular cells and the consequent replacement of the extracellular matrix. The progressive depletion of renal tubular cells results from apoptosis and necroptosis; however, the relative significance of each of these cell death mechanisms at different stages during the progression of chronic kidney disease (CKD) remains unclear. We sought to explore the mechanisms of renal tubular cell death during the early and intermediate stages of chronic renal damage of subtotal nephrectomied (SNx) rats. The results of tissue histological assays indicated that the numbers of necrotic dying cells and apoptotic cells were significantly higher in kidney tissues derived from a rat model of CKD. In addition, there was a significant increase in necroptosis observed by transmission electron microscopy (TEM) and an increase in the proportion of TUNEL-positive cells in kidney tissues from SNx rats compared with control rats, and necrostatin-1 (Nec-1) could inhibit necroptosis and reduce the proportion of TUNEL-positive cells. More importantly, we observed a significant increase in the incidence of necroptosis compared with apoptosis by TEM in vivo and in vitro and a significant increase in the proportion of TUNEL-positive tubular epithelial cells that did not express caspase-3 compared with those expressing cleaved caspase-3 in vitro. Furthermore, treatment with Nec-1 and zVAD strongly reduced necroptosis- and apoptosis-mediated renal tubular cell death and decreased the levels of blood urea nitrogen and serum creatinine and tubular damage scores of SNx rats. These results suggest that necroptotic cell death plays a more significant role than apoptosis in mediating the loss of renal tubular cells in SNx rats and that effectively blocking both necroptosis and apoptosis improves renal function and tubular damage at early and intermediate stages of CKD.

## Introduction

Chronic kidney disease (CKD) is the final stage of various renal diseases and is now recognized as a significant global public health problem. CKD is responsible for an estimated 8–16% of deaths in the general population [1]and is associated with an increasing mortality rate [2–5]. Tubulointerstitial fibrosis (TIF) is commonly observed in end-stage renal disease, and this pathological presentation is a more reliable indicator of renal function and CKD prognosis than glomerular damage [6–9].

Although the precise mechanisms mediating the pathogenesis of TIF remain unclear, a growing body of evidence indicates that the ongoing loss of renal tubular cells and their replacement by fibroblasts and amorphous fibrous components of the extracellular matrix contributes to TIF[10]. Multiple studies have demonstrated that the depletion of tubular cells by apoptosis progressively increases over the course of CKD and contributes to the tubular atrophy and renal fibrosis associated with the progression of CKD in experimental and clinical settings [11–14]. We previously demonstrated that necroptosis contributed to the progressive depletion of renal tubule cells, thereby promoting the progression of tubular atrophy and CKD in rats that had undergone subtotal nephrectomy (SNx). Moreover, treating SNx rats with necrostatin-1 (Nec-1), a specific inhibitor of RIP1, blocked necroptotic renal cell death [15,16], thereby improving renal function and alleviating renal fibrosis. However, the relative significance of apoptosis and necroptosis during different stages of progressive renal tubular cell loss and the interplay between these mechanisms remain unclear. We sought to determine the significance of different modes of cell death promoting the progressive loss of tubular cells and the progression of tubular atrophy and CKD.

## Materials and Methods

### Animals and experimental design

The adult male Sprague-Dawley rats (n = 50) used in this study were obtained from the Experimental Animal Center of Chongqing Medical University. The experimental protocols adhered to the Guidelines for the Care and Use of Laboratory Animals approved by the Institutional Ethics Committee of Chongqing Medical University [Permit No. SCXK (Chongqing) 2007–0001] and the State Science and Technology Commission of China. All rats were housed under standard conditions with a 12-h light/dark cycle at 22±2°C and 55±5% humidity. The animals were fed a standard rodent diet and given free access to water. The 50 animals were randomly assigned to either a SNx group (n = 26) or a control group (n = 24). The rats in the SNx group underwent SNx surgery, and the rats in the control group underwent a sham surgery. Two rats died during the second nephrectomy operation as a result of the anesthesia. The rats in the SNx group were further divided into 1 of 4 sub-groups: a SNx+vehicle group, a SNx+zVAD group, a SNX+Nec-1 group, and a SNx+zVAD+Nec-1 group (n = 6). The rats in the control group were also assigned to 1 of 4 sub-groups: a control+vehicle group, a control+zVAD group, a control+Nec-1 group, and a control+zVAD+Nec-1 group (n = 6).

### The SNx rat model and drug administration

The SNx rat model was established as previously reported by Amann et al. [17] and Piecha et al. [18].After a 7-day adaptation period, the rats were anesthetized via intraperitoneal injection of pentobarbital sodium, and the right kidneys of rats in the SNx group were removed. Seven days later, the upper and lower cortex of the left kidney (approximately 60%-70% of the right kidney by weight) was removed, and 1/3 of the left kidney was preserved. The rats in the control group underwent only renal decapsulation. During the 4th week after the second operation, zVAD (1.0 mg/kg per day)[19](MP Biomedicals, Solon, OH, USA) dissolved in 10% dimethyl sulfoxide (DMSO)(Sigma Aldrich, St. Louis., MO, USA) was administered to rats in the SNx+zVAD group, while Nec-1 (1.65 mg/kg/d)[20](Sigma Aldrich, St. Louis., MO, USA) dissolved in 10% DMSO was administered to rats in the SNx+Nec-1 group, and zVAD (1.0 mg/kg per day) and Nec-1 (1.65 mg/kg per day) were administered to rats in the SNx+zVAD+Nec-1 group. The drug treatment was continued for an additional 4 weeks. Eight weeks after the second surgical intervention, which has previously been reported to be the appropriate time to observe renal pathology in the SNx rat model[16,21], the animals were anesthetized as described above, and blood samples were collected through cardiac puncture, after which the animals were euthanized via cervical dislocation. The blood samples were centrifuged, the plasma was separated to assess the renal function of the rats, and the remaining kidney tissue was resected. One portion of the kidney tissue was fixed in 2.5% glutaraldehyde phosphate buffer (pH 7.4), and another was fixed in 4% phosphate-buffered formaldehyde for morphological assays, TUNEL staining and immunohistochemistry analyses. The remaining kidney tissue was snap-frozen in liquid nitrogen and stored at −80°C prior to protein extraction.

### Renal function

Blood urea nitrogen and serum creatinine levels were used to assess renal function in the rats. These parameters were assayed using an automatic biochemical analyzer (Roche Hx-49, Mannheim, Germany) according to the manufacturer’s protocol.

### Renal morphology

The rats were euthanized and perfused with PBS to remove their blood. One portion of the kidney tissue was fixed in 4% phosphate-buffered formaldehyde and embedded in paraffin. To assess renal tubular epithelial cell morphology in SNx rats, 4-micrometer-thick sections were stained with hematoxylin and eosin (H&E). The morphological changes in the kidney tissue and the presence of renal tubular lesions were evaluated in a double-blind fashion. The tubular damage scores were evaluated using a microscope as previously described by Garber et al. [22].Tubular damage was scored as follows: for tubular dilatation, 0 = no dilation and 1 = dilated tubules; for tubular atrophy, 0 = no atrophy, 1 = signs of atrophy and 2 = apoptosis and desquamation; for intracellular vacuoles, 0 = none, 1 = mild (<10 cells per field of view) and 2 = severe; and for hyaline deposition, 0 = no deposits and 1 = hyaline deposits. The total index score for each rat was expressed as the mean value of all of the scores obtained.

### TUNEL assay

Cell death was examined in kidney sections using an in situ cell death detection kit (Roche Applied Science, Indianapolis, USA) according to the manufacturer’s protocol. Briefly, the sections were permeabilized with 1% proteinase K for15 min, then rinsed with PBS, incubated with TUNEL reagents for 1 h at 37°C, and washed in PBS for 5 min. Five randomly selected, non-contiguous regions within the junction between the renal cortex and the medulla were evaluated using a microscope. A blinded investigator evaluated the sections and recorded the average number of TUNEL-positive cells.

### Cell culture

The NRK-52E rat renal tubular epithelial cell line was purchased from the Cell Bank of the Chinese Academy of Sciences (Shanghai, China).The cells were cultured in DMEM/F12 medium (Gibco Life Technologies, Carlsbad, CA, USA) supplemented with 10% fetal bovine serum (Gibco Life Technologies, Carlsbad, CA, USA), 100 U/mL penicillin and 100 U/mL streptomycin (HyClone Laboratories, Inc., Logan, UT, USA). All cells were cultured in a humidified incubator with 5% CO_2_ at 37°C.

### Immunofluorescence analysis of cleaved caspase-3 and in situ fluorescent TUNEL staining

Synchronized NRK-52E cells were cultured in serum-free medium for 24h prior to each experiment, then pretreated with DMSO (1%), Nec-1(30 mmol/L), zVAD(25 mmol/L), or Nec-1(30 mmol/L) with zVAD(25 mmol/L) for 30 min at 37°C.The cells were subsequently treated with TNF-a(100 ng/mL) for 24h[20]. Following treatment, the cells were transferred to adhesive polylysine-coated glass slides and fixed in 4% paraformaldehyde at room temperature for 20-30 min. The slides were then washed 3 times with 0.1 M PBS and incubated with antigen unmasking solution supplemented with 20 μg/mL proteinase K for 15 min at 37°C. The slides were then washed with 0.1%Triton X-100 and blocked with 10% goat serum. After the blocking solution was removed, the slides were incubated with a rabbit polyclonal antibody against cleaved caspase-3 (#9664P, Cell Signaling Technologies, Danvers, MA)(1:100) overnight at 4°C and subsequently incubated with Alexa Fluor 594-conjugated goat anti-rabbit IgG (Beyotime, Jiangsu, China). The samples were next washed 3 times with PBS, incubated with the in situ cell death detection kit reagents (Fluorescein, Roche, Basel, Switzerland) according to the manufacturer’s instructions, and counterstained with 4', 6-diamidino-2-phenylindole (DAPI). The total number of TUNEL-positive cells and the number of TUNEL-positive cells that expressed or did not express cleaved caspase-3were counted in 3–5 non-contiguous fields of view from each specimen in images obtained using a laser scanning confocal microscope (LEICA TCS SP2, Wetzlar, Germany).The proportions of TUNEL-positive cells with cleaved caspase-3-positive or cleaved caspase-3-negative were calculated by a pathologist blinded to the experimental conditions.

### Transmission electron microscopy (TEM)

After the animals were sacrificed, 1-mm^3^ renal tissue fragments were fixed in 2.5% glutaraldehyde phosphate buffer (pH 7.4) overnight at 4°C. Synchronized NRK-52E cells were pretreated for 30 min at 37°C with DMSO(1%), Nec-1(30 mmol/L), zVAD(25 mmol/L), or Nec-1(30 mmol/L) with zVAD(25 mmol/L) and subsequently treated with TNF-a(100 ng/mL) for 24 h. The cells were then prefixed with 2.5% glutaraldehyde for 2 h, rinsed 3 times in 0.1 mol/L PBS (pH 7.4), and post-fixed in 2% osmium tetroxide. Next, the fixed renal tissue fragments and cells were dehydrated and embedded in epoxy resin. Finally, the samples were sectioned and stained with uranyl acetate and lead citrate, and the cells were evaluated using a transmission electron microscope (Hitachi-7500, Japan) as previously described [16].

### Western blot analysis

The kidney tissue specimens frozen in liquid nitrogen were pulverized using a mortar and pestle and lysed in ice-cold RIPA buffer (Beyotime, Jiangsu, China). The total protein concentration was determined using an Enhanced BCA Protein Assay kit (Beyotime, Jiangsu, China) according to the manufacturer’s instructions. Protein samples were mixed with 5×sodium dodecyl sulfate (SDS)-polyacrylamide gel electrophoresis (PAGE) sample loading buffer, boiled for 5 min, and separated via SDS-PAGE. The proteins were then transferred to a polyvinylidene difluoride (PVDF) membrane (EMD Millipore, USA). These membranes were blocked with a 5% non-fat dry milk solution for 1.5 h at room temperature and incubated overnight at 4°C with the following primary antibodies: polyclonal rabbit anti-RIP3 (ab62344, Abcam, Inc., Cambridge, MA, USA) (1:1000 dilution), polyclonal goat anti-caspase-3 (Sc-1225, Santa Cruz Biotechnology Inc., Dallas, Texas, USA) (1:500 dilution), and monoclonal rabbit anti-β-actin (1854–1,Santa Cruz Biotechnology, CA, USA) (1:1000 dilution). Western blot assays were conducted using conventional methods as previously described [23]. The protein bands of interest were visualized using an ECL detection kit (Beyotime, Jiangsu, China). Protein expression was quantified through densitometry analysis with a Bio-Image Analysis System (Bio-Rad, Hercules, CA, USA). The expression ratio of each target protein was normalized to the expression of β-actin.

### Immunohistochemistry

Sections of formaldehyde-fixed, paraffin-embedded kidney tissue samples with a thickness of 4 μm were mounted on polylysine-coated slides. After deparaffinisation to distilled water, the sections were heated in a target-unmasking fluid (Beyotime, Jiangsu, China) for antigen retrieval and incubated with 0.3% hydrogen peroxide in methanol to eliminate endogenous peroxidase activity. Next, the slides were blocked with 10% goat serum and incubated overnight at 4°C with the following primary antibodies: polyclonal rabbit anti-RIP3 (ab62344, Abcam, Inc., Cambridge, MA, USA) (1:200 dilution) and polyclonal rabbit anti-cleaved caspase-3 (#9664P, Cell Signaling Technologies, Danvers, MA) (1:100 dilution). Then, the slides were treated with a goat anti-rabbit secondary antibody for 30 min. Finally, the sections were counterstained with hematoxylin. Samples that had not been incubated with the primary antibody were used as negative controls. Five randomly selected, non-contiguous regions within the junction between the renal cortex and the medulla were analyzed using a microscope. The proportion of tubular epithelial cells that expressed each marker was calculated by a blinded pathologist.

### Statistical analysis

All data were expressed as the mean±SEM. Each experiment was conducted a minimum of 3 times, and the results of 1 representative experiment are shown in the figures. SPSS 17.0 statistical software (SPSS Inc., IL, USA) was used for statistical analyses. Multiple group comparisons were performed via one-way ANOVA, and the Bonferroni method was used for the comparison of means. The Mann-Whitney test was employed to compare the incidence of necroptosis and apoptosis in the tubular epithelial cells treated with TNF-a alone. P<0.05 was considered statistically significant.

## Results

### 1. Renal function

**Fig 1:**
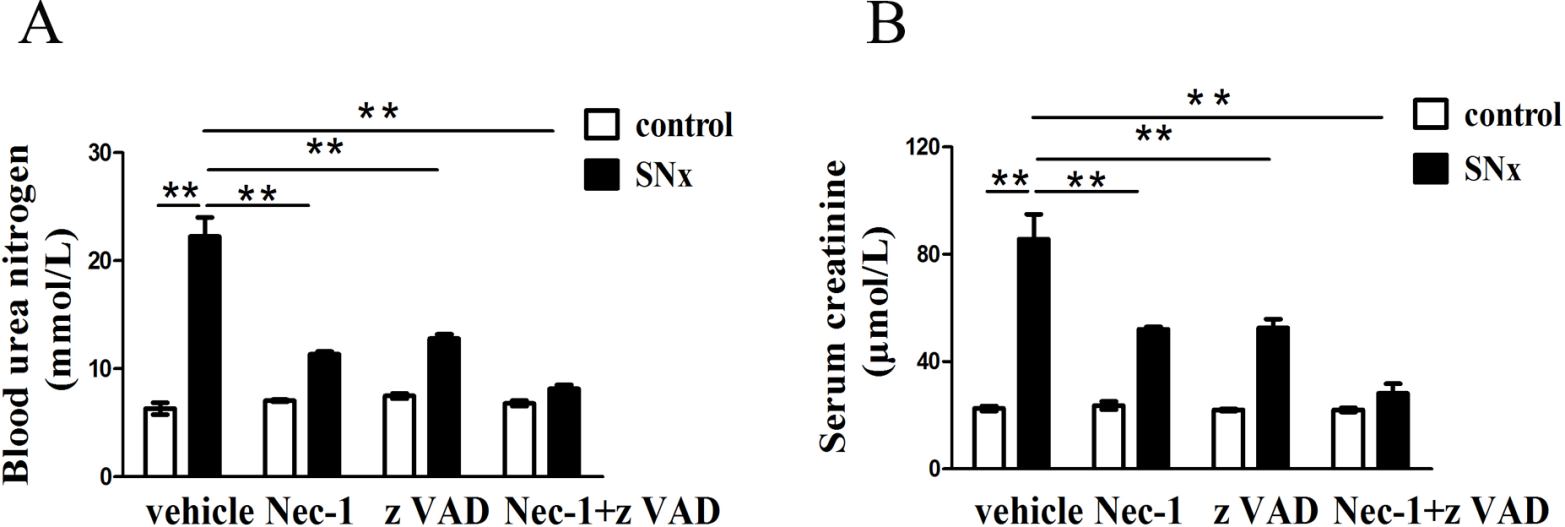
Levels of blood urea nitrogen and serum creatinine. The levels of blood urea nitrogen (A) and serum creatinine (B) were decreased in rats treated with Nec-1, zVAD or both Nec-1 and zVAD compared with the control group.(n = 6 rats per group, **p<0.01 versus the SNx+vehicle group). SNx: subtotal nephrectomy.

We assessed the renal function of the rats at 8 weeks after they had undergone SNx surgery. This time point was chosen because TIF with segmental sclerosis and the greatest extent of renal tubular epithelial cell death is observed at 8 weeks after rats are subjected to SNx surgery [16,24]. Compared with the control animals, blood urea nitrogen and serum creatinine levels were increased in SNx rats. Treating SNx rats with Nec-1 or zVAD alone for 4 weeks improved renal function, as demonstrated by the significant decrease in blood urea nitrogen and serum creatinine levels. The greatest improvements were observed in rats that received combined treatment with Nec-1 and zVAD (Fig 1). However, no significant differences were observed in the sham-operated groups (Fig 1).

### 2. Morphological changes in renal tubular cells

**Fig 2:**
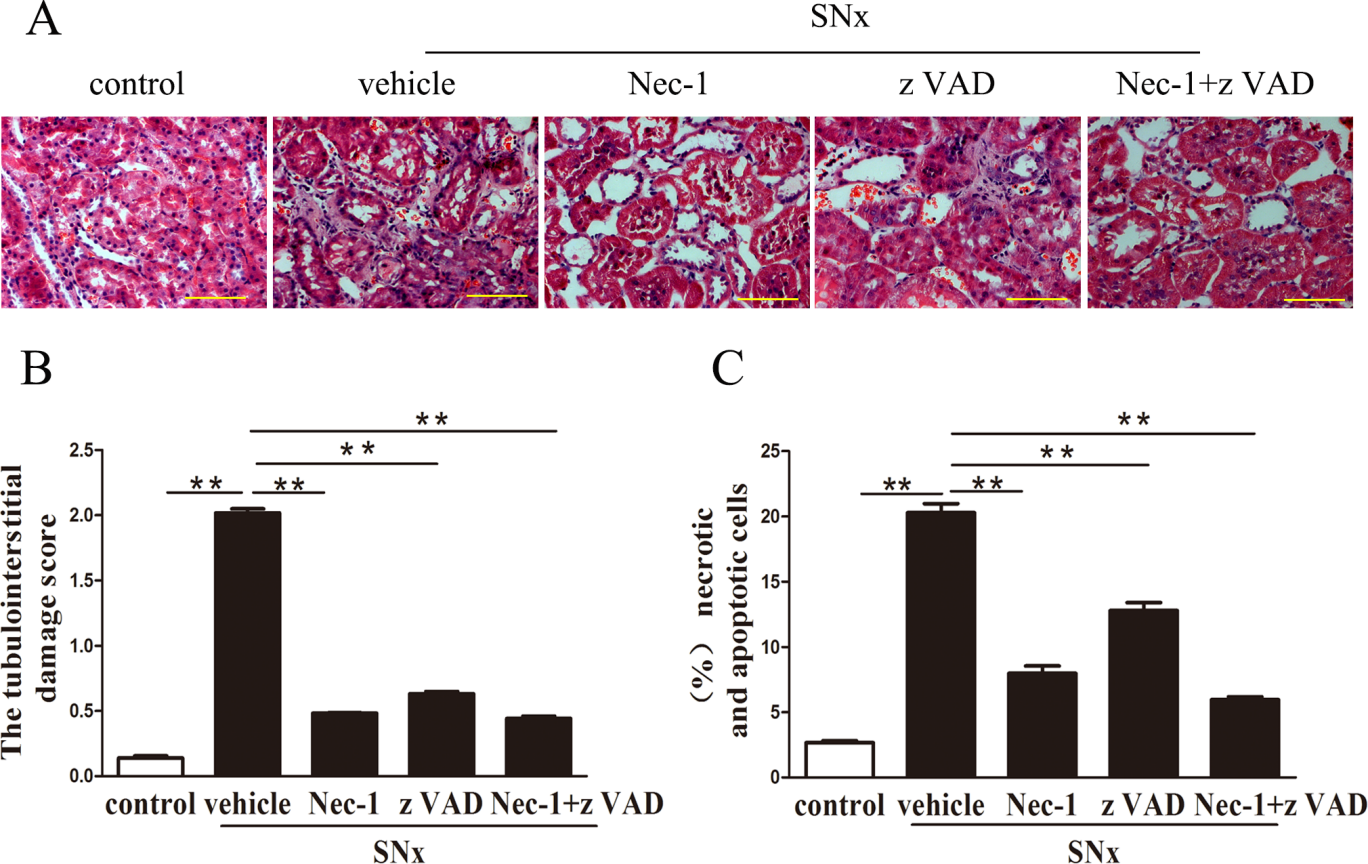
Morphological changes in renal tubules. (A) Representative images of H&E-stained kidney tissue sections exhibiting tubular dilatation, atrophy, desquamation of epithelial cells and hyaline deposition in the tubular lumen from the SNx rats, Scale bar, 50μm.(B) Tubular damage scores were decreased in rats treated with Nec-1,zVAD, or both Nec-1 and zVAD. (C)The proportion of necrotic dying cells and apoptotic cells. (n = 6 rats per group, **p<0.01 versus the SNx+vehicle group). SNx: subtotal nephrectomy.

H&E-stained kidney tissues form the SNx+vehicle group exhibited signs of tubular damage, including tubular dilatation, atrophy, desquamation of epithelial cells, and hyaline deposition in the tubular lumen; these features were not observed in the control group (Fig 2A). The tubular damage scores and numbers of necrotic dying cells and apoptotic cells were significantly higher in kidneys from the SNx+vehicle group than in those from the control group. Treatment with Nec-1, zVAD or Nec-1and zVAD significantly ameliorated the above pathological lesions and inhibited the increase in tubular damage scores and the percentage of necroptotic and apoptotic cell death in rats subjected to SNx surgery, and this effect was most robust in animals treated with both Nec-1and zVAD (Fig 2B and 2C). In addition, tubular epithelial cell vacuolization was observed only in the control group (Fig 2A).

### 3. Detection of renal tubular epithelial cell death using TEM in vivo and in vitro

**Fig 3:**
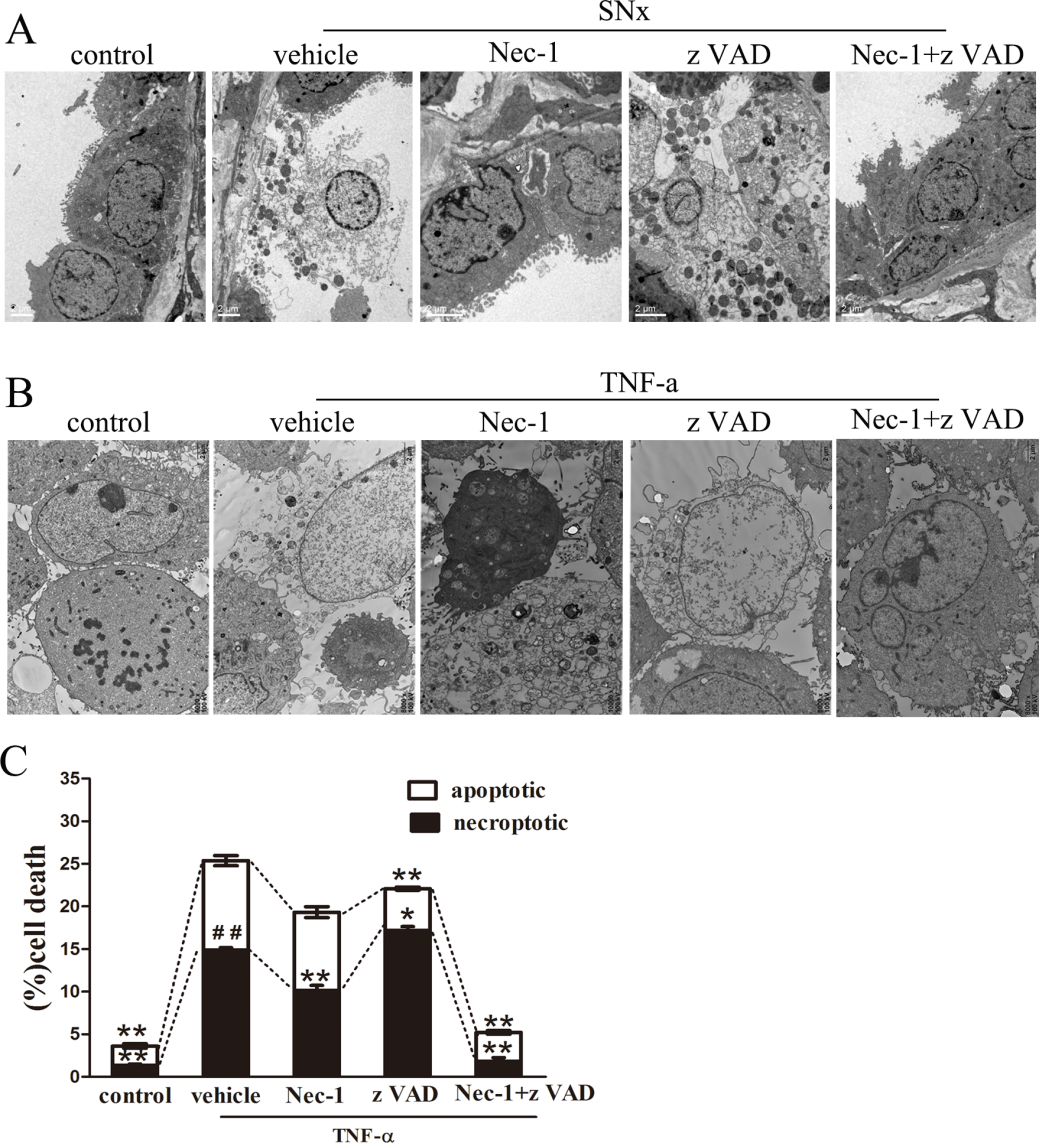
Necroptotic renal tubular epithelial cells were evaluated in vivo and in vitro using TEM. (A) TEM photomicrographs of renal tubular epithelial cells from sham-operated rats and SNx rats treated with vehicle, Nec-1, zVAD, or Nec-1 and zVAD. Scale bar, 2μm. (B) TEM photomicrographs of cultured renal tubular epithelial cells pretreated for 30 min with DMSO (1%), Nec-1(30 mmol/L), zVAD (25 mmol/L), or Nec-1(30 mmol/L) and zVAD(25 mmol/L) and subsequently treated with TNF-a (100 ng/mL) for 24 h. (Scale bar, 2μm). (C) Quantification of the necroptotic incidence and apoptotic incidence in tubular epithelial cells in vivo. (*p<0.05, **p<0.01versus the tubular epithelial cells treated with only TNF-a, ##p < 0.01 necroptotic incidence versus apoptotic incidence in tubular epithelial cells treated with only TNF-a). SNx: subtotal nephrectomy.

To further characterize the morphological characteristics of dead cells at the ultrastructural level, we used TEM to analyze renal tubular epithelial cells from remnant kidney samples. Consistent with the results of the H&E staining assays, TEM analysis revealed that necroptotic renal tubular epithelial cell death in the SNx+vehicle group and the SNx+zVAD group was characterized by an expansion of the cell volume, organelle swelling, plasma membrane rupture, loss of cell organelle contents and extensive intracellular vacuole formation (Fig 3A). Cells hrinkage, nuclear condensation and other characteristic features of apoptosis were occasionally observed in kidneys derived from the SNx+vehicle group. Furthermore, in the kidneys of rats treated with Nec-1 or Nec-1 in combination with zVAD, volume expansion was observed in relatively few renal tubular cells (Fig 3A). In addition, tubular epithelial cell vacuolization was observed only in the control groups.

In addition, we evaluated NRK-52E cells in vitro using TEM. The analysis demonstrated that the incidence of necroptosis and apoptosis was significantly higher in NRK-52E cells stimulated with TNF-α(10.5%±0.60% apoptotic cells, 14.87%±0.29% necrotic cells) than in control cells treated with PBS(2.26%±0.27% apoptotic cells, 1.36%±0.15% necrotic cells). Notably, the incidence of necroptosis was greater than that of apoptosis for cells stimulated with TNF-α (10.5%±0.60% apoptotic cells, 14.87%±0.29% necrotic cells Fig 3B and 3C). Compared with the TNF-α-only treatment, zVAD significantly decreased apoptosis and increased necroptosis in TNF-α-stimulated NRK-52E cells(4.89%±0.19% apoptotic cells, 17.19%±0.43% necrotic cells). Furthermore, Nec-1 treatment blocked necroptosis, but not apoptosis, in cells stimulated with TNF-α (9.17%±0.64% apoptotic cells, 10.14%±0.59% necrotic cells, Fig 3B and 3C), and treatment with Nec-1 and zVAD significantly inhibited both apoptosis and necrosis in these cells(3.38%±0.20% apoptotic cells, 1.83%±0.41% necrotic cells).

These findings in vitro and in vivo indicated that the primary mode of renal tubular epithelial cell death was necroptosis, not apoptosis.

### 4. Detection of dead renal tubular epithelial cells using TUNEL staining in vivo and in vitro

**Fig 4:**
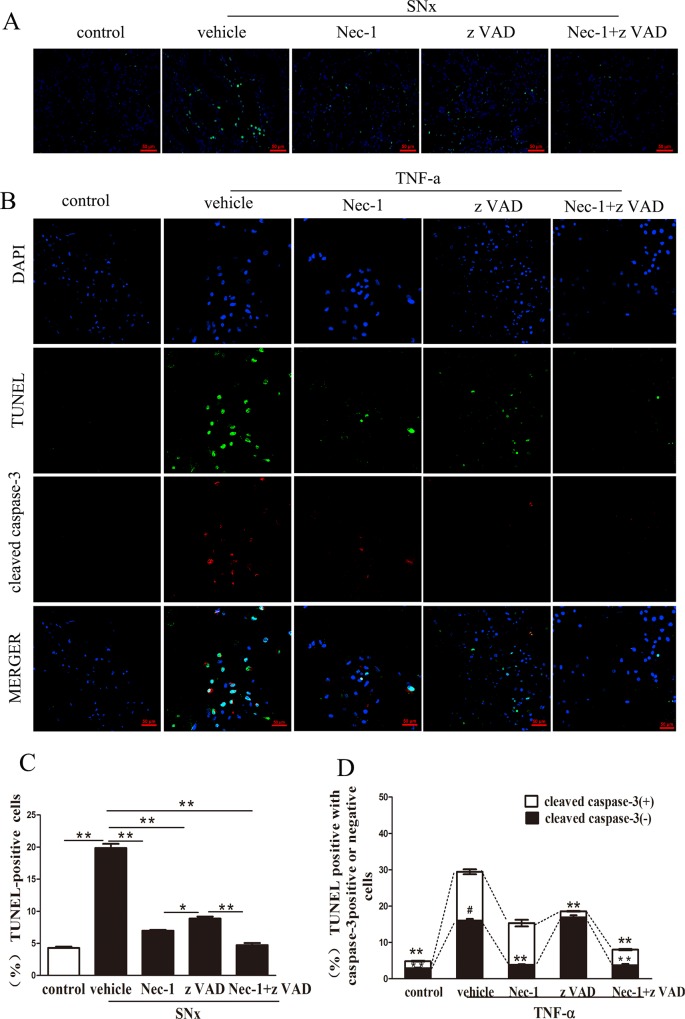
Detection of dead renal tubular epithelial cells using TUNEL staining in vivo and in vitro. (A) TUNEL (green) and DAPI (blue) staining in renal tubular epithelial cells in sham-operated rats and SNx rats treated with vehicle, Nec-1, zVAD, or Nec-1 and zVAD. Scale bar, 50μm. (B) Immunofluorescence staining of cleaved caspase-3 (red),TUNEL staining (green) and DAPI (blue) staining in renal tubular epithelial cells pretreated for 30 min with DMSO (1%), Nec-1(30 mmol/L), zVAD(25 mmol/L), or Nec-1(30 mmol/L) and zVAD(25 mmol/L) and subsequently treated with TNF-a (100 ng/mL) for 24 h.(Scale bar, 50μm). (C) Quantification of TUNEL-positive cells in sham-operated rats and SNx rats treated with vehicle, Nec-1, zVAD, or Nec-1 and zVAD (n = 6 rats per group, **p<0.01 versus the SNx+vehicle group). SNx: subtotal nephrectomy. (D) Quantification of TUNEL-positive, cleaved caspase-3-positive cells and TUNEL-positive, cleaved caspase-3-negative cells in vivo (*p<0.05, **p<0.01versus the tubular epithelial cells treated with TNF-a alone, ##p <0.01 necroptotic incidence versus apoptotic incidence in tubular epithelial cells treated with TNF-a alone). SNx: subtotal nephrectomy.

To further evaluate the mechanisms of cell death in the SNx rats, we examined the remnant kidney tissue cells for the presence of fragmented DNA using TUNEL staining assays. TUNEL-positive cells were observed in renal tubules, with relatively higher levels being observed in the proximal renal tubule (Fig 4A). The proportion of TUNEL-positive cells in the kidneys from the SNx+vehicle group was higher than in the control group (Fig 4C). Treatment with zVAD, Nec-1, or Nec-1 in combination with zVAD significantly reduced the total number and the proportion of TUNEL-positive cells, and the greatest reduction was observed in the combination treatment group (Fig 4A and 4C).

To further distinguish the modes of renal tubular cell death, we conducted cleaved-caspase-3 immunostaining and fluorescent TUNEL staining assays on NRK-52E cells (Fig 4B and 4D). The proportion of TUNEL-positive cells with Caspase-3 positive or negative was significantly greater in the TNF-α-stimulated cells(13.45%±0.69% TUNEL-positive with Caspase-3 positive cells, 16.00%±0.46% TUNEL-positive with Caspase-3 negative cells)than in the control cells pretreated with PBS (1.91%±0.13% TUNEL-positive with Caspase-3 positive cells, 2.92%±0.15% TUNEL-positive with Caspase-3 negative cells). In the cells pretreated with zVAD and TNF-α, the proportion of TUNEL-positive, cleaved caspase-3-positive cells decreased, and the proportion of TUNEL-positive, cleaved caspase-3-negative cells did not significantly increased (1.69%±0.10% TUNEL-positive with Caspase-3 positive cells, 16.89%±0.59% TUNEL-positive with Caspase-3 negative cells). In the cells pretreated with TNF-α and Nec-1, the proportion of TUNEL-positive, cleaved caspase-3-negative cells decreased, but the proportion of TUNEL-positive, cleaved caspase-3-positive cells was unaffected (11.47%±0.92% TUNEL-positive with Caspase-3 positive cells, 3.83%±0.19% TUNEL-positive with Caspase-3 negative cells). Furthermore, in the cells pretreated with TNF-α in combination with both Nec-1 and zVAD, the total proportion of TUNEL-positive cells significantly decreased(4.27%±0.16% TUNEL-positive with Caspase-3 positive cells, 3.75%±0.33% TUNEL-positive with Caspase-3 negative cells). More importantly, we observed a significant increase in the proportion of TUNEL-positive tubular epithelial cells that did not express caspase-3 compared with those expressing cleaved caspase-3 in the TNF-α-stimulated cells(13.45%±0.69% TUNEL-positive with Caspase-3 positive cells, 16.00%±0.46% TUNEL-positive with Caspase-3 negative cells), consistent with the results of TEM assays in vitro.

In vivo and in vitro TUNEL staining indicated that the loss of renal tubular epithelial cells was mediated by necroptotic cell death, apoptosis, and the interplay between these two mechanisms.

### 5. RIPK3 and caspase-3 protein levels in SNx rat kidney tissue

**Fig 5:**
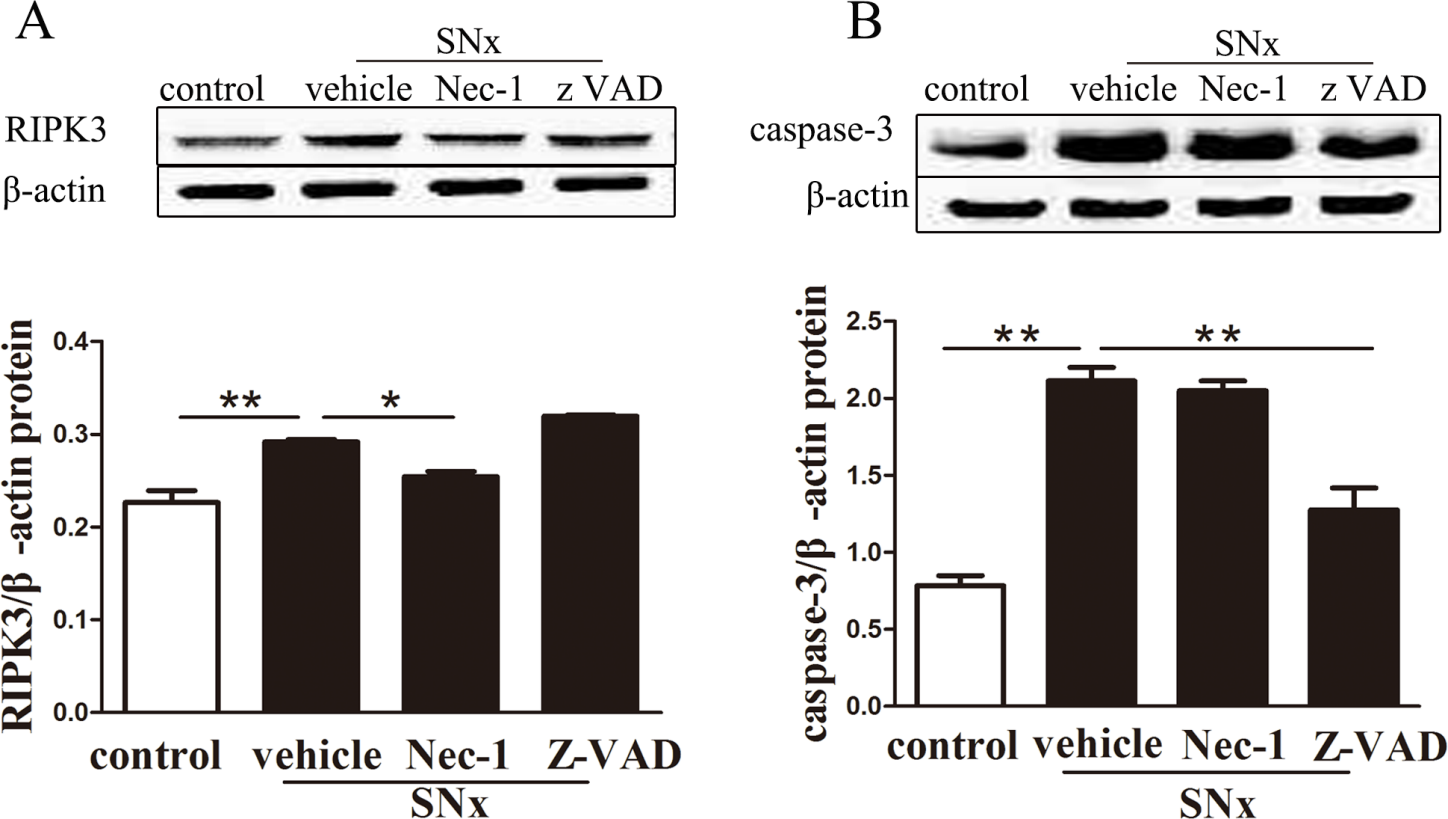
Western blot analysis of RIPK3 and caspase-3 protein levels in the remaining kidney tissue of SNx rats. RIPK3 (A) and caspase-3(B) protein expression in the remaining kidney tissue of sham-operated rats and SNx rats treated with vehicle, Nec-1, or zVAD. (n = 6 rats per group, **p<0.01 versus the SNx+vehicle group). SNx: subtotal nephrectomy.

To further investigate the dynamics of renal cell death at the molecular level, the protein levels of RIPK3 and caspase-3 in the kidney tissues of SNx rats were evaluated using western blotting and immunohistochemistry staining. Western blot analysis demonstrated that RIPK3 and caspase-3 protein levels were increased in kidney tissues from SNx rats compared with sham-operated rats (Fig 5).Treatment with Nec-1 significantly reduced RIPK3 protein levels in SNx rats. In contrast, zVAD treatment increased RIPK3 protein levels (Fig 5A). In addition, caspase-3 levels were decreased in SNx rats treated with zVAD but were unaffected in SNx rats treated with Nec-1(Fig 5B).

**Fig 6:**
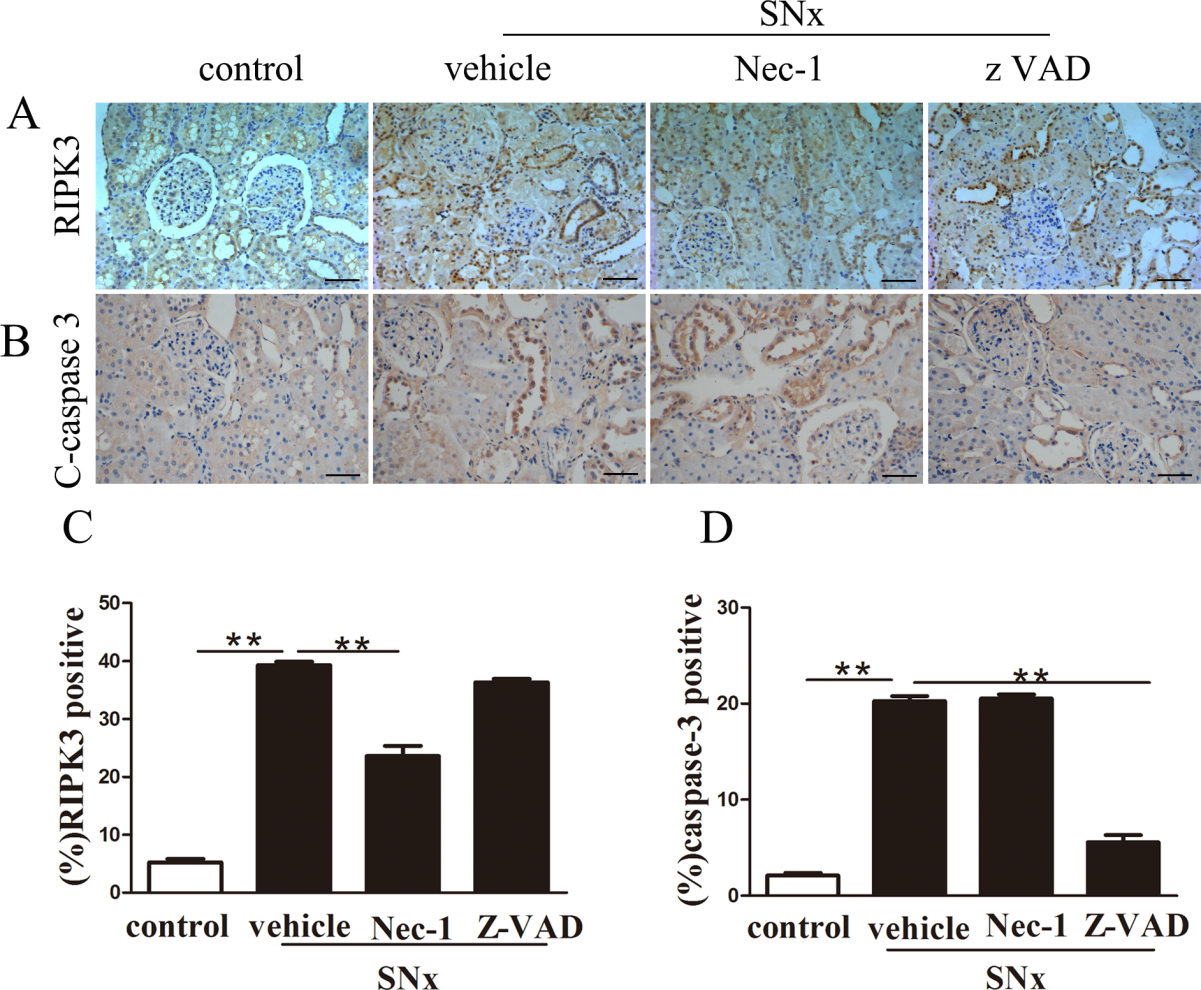
Immunohistochemistry analysis of RIPK3 and cleaved caspase-3 protein expression in the remaining kidney tissues of SNx rats. RIPK3 (A) and cleavedcaspase-3(B) in sham-operated rats and SNx rats treated with vehicle, Nec-1, or zVAD. Scale bar, 50μm. Semi-quantitative analysis of RIPK3 (C)and cleaved caspase-3(D) expression in renal tubular epithelial cells derived from SNx rats treated with vehicle, Nec-1or zVAD. (n = 6 rats per group, **p<0.01 versus the SNx+vehicle group). SNx: subtotal nephrectomy.

Immunohistochemistry staining revealed that RIPK3 and cleaved caspase-3 localized to the cytoplasm of renal tubular epithelial cells, and RIPK3 and caspase-3 levels were particularly high within regions of tubular injury (Fig 6A and 6B).Immunostaining assays also revealed that the proportion of RIPK3-positive, caspase-3-positive cells was significantly greater in SNx rats than in sham-operated rats (Fig 6C and 6D). In contrast, the proportion of RIPK3-positive cells was substantially decreased in the SNx rats treated with Nec-1and increased in the SNx rats treated with zVAD (Fig 6C). Moreover, caspase-3 immunostaining was greatly decreased in cells treated with zVAD but was unaffected in the SNx group treated with Nec-1 (Fig 6D). These data were consistent with the results of the western blot analysis.

## Discussion

An increasing body of evidence suggests that necroptosis, similar to apoptosis, is a highly regulated process of programmed cell death by a distinct signal transduction pathway [25–27]. Previous studies have demonstrated that apoptotic and necroptotic cell death are the important mechanisms that mediate renal tubular cell depletion and CKD progression in the SNx rat model [16,25,26]. However, the dynamics of cell death during the early and intermediate stages of CKD have remained unclear. In the present study, we evaluated renal tubular cell death in the SNx rat model, as this model recapitulates the progression of CKD in humans. SNx rats exhibit TIF with segmental sclerosis by week 8 after surgery, and these defects persist through week 12 to week 16[18]. The diffuse glomerular sclerosis and TIF occurs and SNx rats frequently die of uremia. The highest incidence of renal tubular cell death in the remaining kidney tissue of SNx rats is observed 8 weeks after nephrectomy [21,24]. Therefore, 8 weeks after SNx appears to be the appropriate time point at which to analyze renal tubule cell depletion and renal TIF. In the study, the kidney tissues of SNx rats were characterized by tubular interstitial fibrosis, as demonstrated by the presence of tubular dilatation, atrophy, and epithelial cell desquamation. Rats in the SNx+vehicle group exhibited signs of renal impairment and higher tubular damage scores than the control group. These data indicate that the SNx animal model analyzed in this study mimics the progression of CKD.

Due to the absence of distinct markers of necroptosis and apoptosis, it is difficult to distinguish between these mechanisms in dying cells. Therefore, we evaluated necroptotic cell death in vitro and in vivo using a variety of methods. First, the morphological characteristics of necroptotic dying cells (intact nuclei) and apoptotic dying cells (nuclear condensation) versus cells with hyperchromatic or pyknotic nuclei (well-represented cells undergoing necrosis or apoptosis, as emphasized by Elston and Ellis[27]) were evaluated in kidney tissue sections stained with H&E. Our histological analysis revealed that renal tubular cell loss was mediated by necroptotic and apoptotic cell death in the SNx rats. Treatment with Nec-1 or zVAD significantly inhibited cell death, and treatment with both Nec-1 and zVAD was associated with the strongest inhibition of cell death. These observations further demonstrated that both necroptotic cell death and apoptosis mediated renal tubular cell death.

Because H&E staining is often supplemented with electron microscopic pictures to illustrate the morphological characteristics of apoptotic and necrotic dying cells [28], we further analyzed the morphological features of renal tubular cells at the ultrastructural level using TEM. Extensive necroptotic cell death was observed in SNx rats treated with vehicle or zVAD, and treatment with Nec-1 improved the changes associated with necroptosis at the ultrastructural level. These findings are consistent with the histological analysis of kidney sections stained with H&E. These results further confirm that necroptotic cell death is the primary mechanism mediating the loss of renal tubular epithelial cells in the early and intermediate stages of chronic renal damage in SNx rats. Interestingly, we observed few apoptotic renal tubular epithelial cells in TEM images of SNx rats. A likely explanation for this finding is that few tubular cells undergo apoptosis in the stages we evaluated. Another possibility is that the methods we used were not sufficiently sensitive to detect apoptosis in the evaluated tissue specimens. To further confirm that necroptosis was the primary mechanism of renal tubular cell death, we compared the proportions of necroptotic and apoptotic renal tubular epithelial cells using TEM in vitro. The incidence of necroptosis was greater than the incidence of apoptosis in cells stimulated with TNF-α in vitro. These findings are consistent with the findings of the TEM analysis in vivo.

While TUNEL staining has long been considered the gold standard in apoptosis assays, Grasl-kraupp B et al. reported that some necrosis can also generate DNA fragments that react with the TUNEL reaction solution [29,30]. Therefore, TUNEL-positive cells might not necessarily be indicative of apoptotic death cell. We found that the proportion of TUNEL-positive cells in kidney tissues from SNx rats treated with Nec-1was lower than in tissues from SNx rats treated with zVAD, consistent with the TEM analyses. The results might be caused by the fact that necroptotic cell death is the primary mechanism mediating cell death in the renal tubular cells of SNx rats at 8 weeks after surgery.

Another possibility reason is that treatment with zVAD alone might aberrantly increase the incidence of necroptotic renal tubular cell, consistent with the important characteristics of necroptosis[31]. Therefore, we speculate that necroptotic cell death is the primary mechanism mediating renal tubular cell death in SNx rats at 8 weeks after surgery, or necroptotic cell death is the primary mechanism when apoptosis is blocked. These results are similar to the findings of a renal ischemic/reperfusion injury study reported by Andreas Linkermann et al. [20].

To further distinguish the mode of cell death in TUNEL-positive cells, we conducted TUNEL assays in combination with anti-cleaved caspase-3 immunostaining assays in vitro. Typically, cells that are TUNEL-positive and cleaved caspase-3-negative are considered necroptotic cells, while cells that are TUNEL-positive and cleaved caspase-3-positive are considered apoptotic cells [32–34]. In the present study, this population of TUNEL-positive, active caspase-3-negative renal tubular cells (necroptotic renal tubular cells) decreased in cells treated with Nec-1 and increased in cells treated with zVAD, thus suggesting that necroptotic cell death mediated the observed renal tubular cell loss. More importantly, we observed a greater proportion of necroptotic renal tubular cells than apoptotic renal tubular cells (TUNEL-positive and cleaved caspase-3-positive renal tubular cells) in the TNF-α-stimulated cells, consistent with the results of TEM assays in vitro, thus indicating that necroptotic cell death, rather than apoptosis, played a more important in renal tubular cell loss.

RIPK3 is a key regulator of the necroptotic cell death pathways and can be recruited to the necrosome complex via a direct interaction between the RHIM domains of RIPK1 and RIPK3 [28,34]. Our previous studies, as well as the findings of this study, indicate that RIPK3 protein levels are enhanced in kidney tissues from SNx rats and that this effect is inhibited by Nec-1 but not by zVAD. These findings were obtained using a variety of methods, including western blot analysis, immunohistochemistry staining, and RIPK1 and RIPK3 colocalization assays. Together, these data suggest that RIPK3-mediated necroptotic cell death plays a role in renal tubular cell depletion.

In addition, blocking necroptosis and/or apoptosis effectively improved renal function and tubular lesion in the early and intermediate stages of CKD of SNx rats (8 weeks after SNx surgery).The greatest therapeutic effect was observed when both mechanisms were inhibited by combination treatment with Nec-1 and zVAD.

## Conclusions

In summary, necroptotic cell death is the primary mechanism mediating the loss of renal tubular cells in rats observed at 8 weeks after SNx surgery, and combined inhibition of necroptosis and apoptosis can improve renal function and tubular damage during early and intermediate stages of chronic renal damage in SNx rats.

## Supporting Information

S1 Fig PAS staining. (TIF)

S1 File Blot original figures. (ZIP)
