# Supplementary figures and images for: RIPK3-Mediated Necroptosis and Apoptosis Contributes to Renal Tubular Cell Progressive Loss and Chronic Kidney Disease Progression in Rats
Source: PLoS One. 2016 Jun 9;11(6):e0156729. doi: 10.1371/journal.pone.0156729 (PMC4900656; doi:10.1371/journal.pone.0156729)

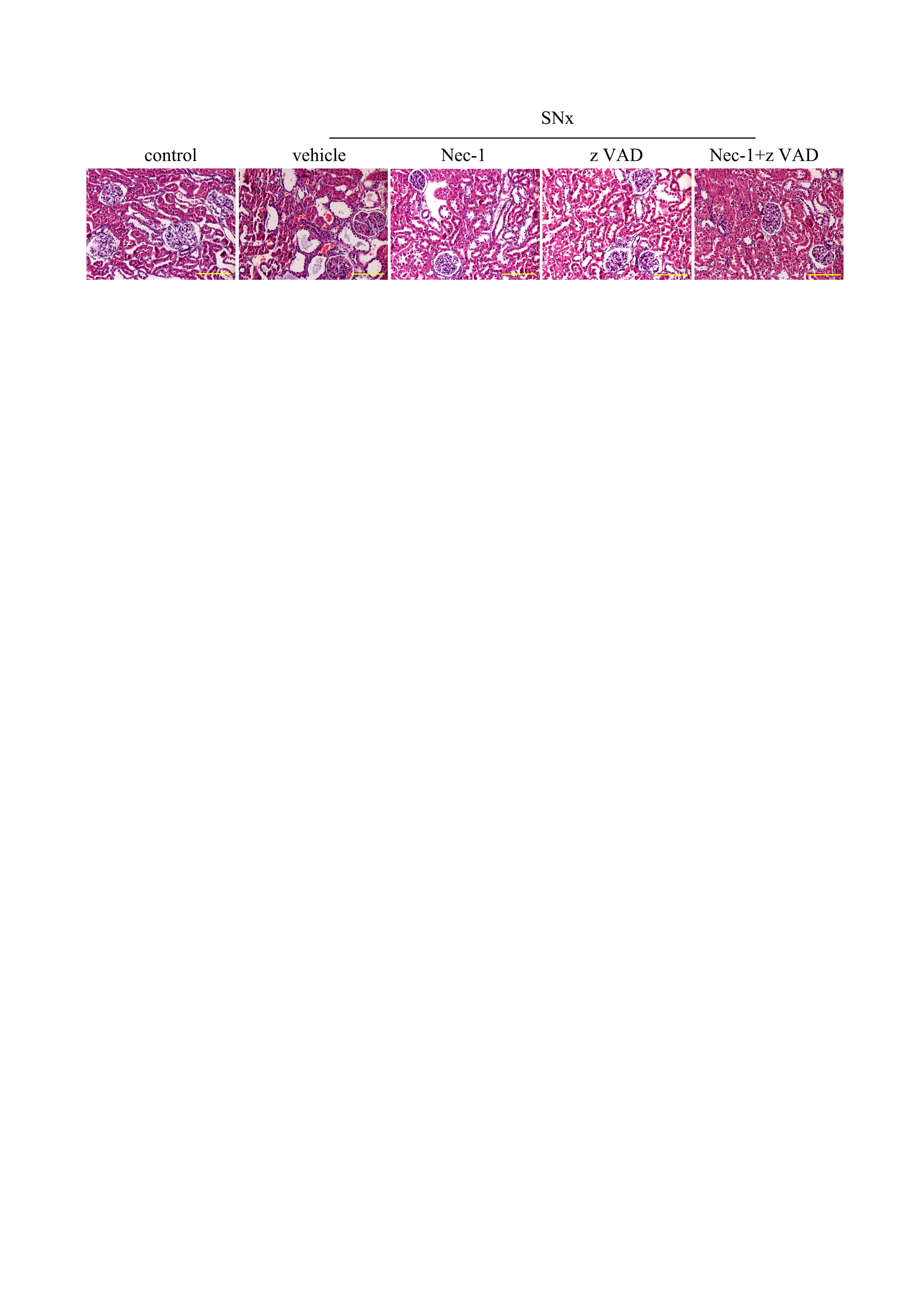

Supplement: S1 Fig — (TIF) [file pone.0156729.s001.tif]

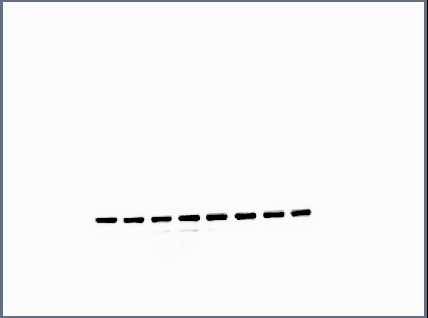

Supplement: S1 File — (ZIP) [file pone.0156729.s002.zip › 2015-07-15 ACTIN 05.jpg]

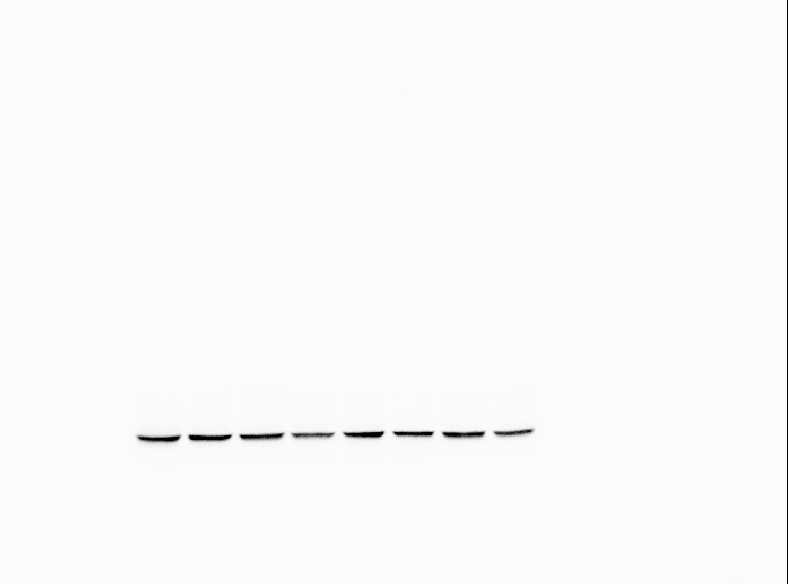

Supplement: S1 File — (ZIP) [file pone.0156729.s002.zip › 2015-07-15 RIP3 06.jpg]

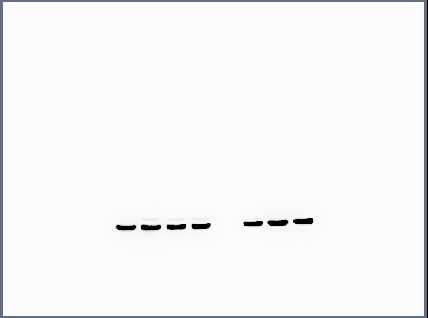

Supplement: S1 File — (ZIP) [file pone.0156729.s002.zip › 2015-07-22 actin 02.jpg]

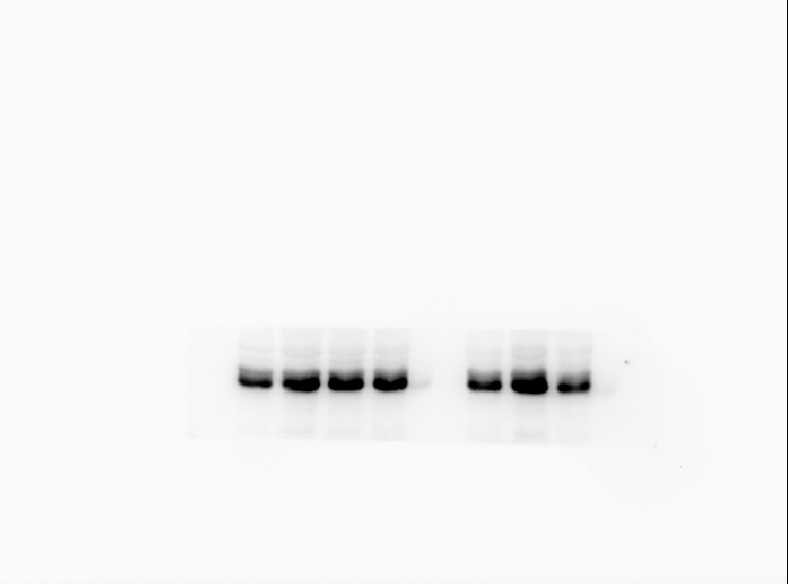

Supplement: S1 File — (ZIP) [file pone.0156729.s002.zip › 2015-07-22 caspase3 05.jpg]
